# Cooperative Adaptation to Establishment of a Synthetic Bacterial Mutualism

**DOI:** 10.1371/journal.pone.0017105

**Published:** 2011-02-16

**Authors:** Kazufumi Hosoda, Shingo Suzuki, Yoshinori Yamauchi, Yasunori Shiroguchi, Akiko Kashiwagi, Naoaki Ono, Kotaro Mori, Tetsuya Yomo

**Affiliations:** 1 Department of Bioinformatic Engineering, Graduate School of Information Science and Technology, Osaka University, Suita, Japan; 2 Department of Frontier Biosciences, Graduate School of Frontier Biosciences, Osaka University, Suita, Japan; 3 Faculty of Agriculture and Life Science, Hirosaki University, Hirosaki, Japan; 4 Exploratory Research for Advanced Technology (ERATO), Japan Science and Technology Agency, Suita, Japan; Texas A&M, United States of America

## Abstract

To understand how two organisms that have not previously been in contact can establish mutualism, it is first necessary to examine temporal changes in their phenotypes during the establishment of mutualism. Instead of tracing back the history of known, well-established, natural mutualisms, we experimentally simulated the development of mutualism using two genetically-engineered auxotrophic strains of *Escherichia coli*, which mimic two organisms that have never met before but later establish mutualism. In the development of this synthetic mutualism, one strain, approximately 10 hours after meeting the partner strain, started oversupplying a metabolite essential for the partner's growth, eventually leading to the successive growth of both strains. This cooperative phenotype adaptively appeared only after encountering the partner strain but before the growth of the strain itself. By transcriptome analysis, we found that the cooperative phenotype of the strain was not accompanied by the local activation of the biosynthesis and transport of the oversupplied metabolite but rather by the global activation of anabolic metabolism. This study demonstrates that an organism has the potential to adapt its phenotype after the first encounter with another organism to establish mutualism before its extinction. As diverse organisms inevitably encounter each other in nature, this potential would play an important role in the establishment of a nascent mutualism in nature.

## Introduction

Mutualism is based on a mutually beneficial interaction between two organisms and is ubiquitous in nature [1], [2], [3], [4], [5], [6]. Mutualisms observed in nature are thought to be the result of adaptation of each organism to the existence of the partner after their first encounter. The genetic origin and trajectory of this adaptation has been investigated via a phylogenetic approach [2], [7], [8]. However, tracing back established mutualisms to their origin is challenging as no intermittent states are defined with which to measure the adaptation in terms of phenotypic traits, population size and local environment [1]. To investigate the environmental conditions required to establish a nascent mutualism, one study reported a synthetically designed mutualism using two species of bacteria [9]. The findings of that study clearly demonstrated the importance of spatially structured environments for the establishment of mutualism, providing proof of principle of natural selection of cooperative behavior that has been proposed by the theoretical studies [10], [11], [12], [13]. These types of experimental studies using microbial ecosystems to test the theories of cooperative systems have recently been reported [14], [15], [16], [17], [18]. Most of these studies focused not on the adaptation of the organisms but on the environmental conditions required for the persistence of cooperative behavior in natural selection.

Some studies have characterized the behavior of organisms in nascent mutualisms. Wintermute *et al.* synthetically designed mutualisms comprising certain pairs of auxotrophs of *Escherichia coli* and found significant metabolic synergy in 17% of 1035 such pairs tested [19], although it was unclear if any adaptation of the bacteria contributed. Shou *et al*. synthetically designed an obligate mutualism composed of two yeast auxotrophs [20], each of which was genetically engineered to overproduce the metabolite essential for the growth of the partner. Both of the auxotrophic strains grew to saturation without the need for external supplementation of their essential metabolites compensating for the auxotrophy. Moreover, they showed adaptation in as little as one hundred generations, where they became capable of growing from diluted cell densities or ceased growth due to weakening of the beneficial interaction. Hillesland *et al*. demonstrated that the growth rate of microorganisms in another synthetic mutualism increased after serial passage, even in the absence of spatially structured environment, while the extent of the adaptation was increased in a spatially structured environment [21]. These adaptations of microorganisms occurred after the establishment of nascent mutualisms, strengthening their interactions.

Can adaptation occur before the establishment of a nascent mutualism, leading to its establishment? Here we show that a strain of bacteria became more beneficial to another strain before their population started growing and establishing a nascent mutualism. Specifically, we synthetically designed an obligate mutualism comprising two auxotrophs of *Escherichia coli*. We show that one of the two auxotrophs, upon encountering the partner strain before their own population growth, adapted by oversupplying the metabolite essential for growth of the partner, which in turn permitted its own growth, leading to the successive growth of both strains. This study therefore shows the potential of organisms to adaptively respond to the first encounter with another organism, which could lead to the establishment of nascent mutualisms. As diverse organisms inevitably encounter each other in nature, this potential would play an important role in the establishment of nascent mutualisms in nature.

## Results

**Figure 1:**
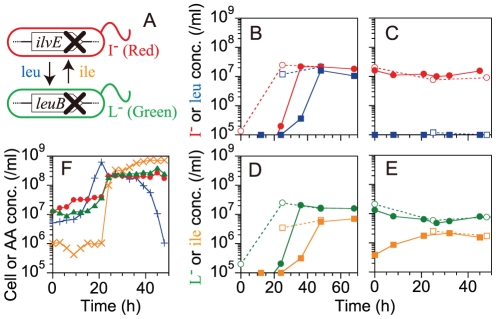
Basic design of the model system and cell growth during synthetic mutualism. (A) Schematic diagram of the synthetic mutualism. Two auxotrophs of *E. coli*, strains I^–^ and L^–^, supply amino acids to each other and potentially establish mutualism, as described in the text. (B–E) Cell growth and nutrient release in the monocultures. The concentration of Leu or Ile is indicated as the density of L^–^ or I^–^ cells which can be produced by the amount of Leu or Ile, respectively. When the amino acid concentration was not detected (under the detection limit 10^5^/ml), we plotted it at 10^5^/ml. (B and C) The time course of the concentration of Leu (blue square) and I^–^ cells (red circle) in monoculture. (B) 10^3^/ml (closed symbol) or 10^5^/ml (open symbol) I^–^ cells were inoculated into minimal media along with 10 µM of Ile. 10 µM of Ile supports the generation of about 10^7^/ml I^–^ cells. (C) 10^7^/ml I^–^ cells were inoculated into minimal media without the addition of Ile. Closed and open symbols represent two independent experiments. (D and E) The time course of Ile (orange square) and L^–^ cells (green circle). (D) 10^3^/ml (closed symbol) or 10^5^/ml (open symbol) of L^–^ cells were inoculated into minimal media along with 10 µM of Leu. 10 µM of Leu was the amount required for the growth of about 10^7^/ml of L^–^ cells. (E) 10^7^/ml of L^–^ cells were inoculated into minimal media without the addition of Leu. Closed and open symbols represent replicates of two individual cultures. (F) The time course of the concentration of amino acids and the cells in coculture: I^–^ (red •), L^–^ (green ▴), Ile (orange ×), Leu (blue +). 10^7^/ml of both I^–^ and L^–^ cells were inoculated into minimal media without the addition of any amino acids.

To create a synthetic model of obligate mutualism, we constructed two different types of nutrient auxotrophs of *E. coli* by genetic recombination (Fig. 1A): an isoleucine (Ile) auxotroph, designated I^–^, labeled with a red-fluorescent protein (*dsred.T3*), and a leucine (Leu) auxotroph, L^–^, labeled with a green-fluorescent protein (*gfpuv5*) [22] (see Methods). We were able to distinguish these two strains by flow cytometry (FCM). In minimal medium without amino acid supplements, neither strain was able to grow in monoculture. However, in coculture, if the two strains supplied a sufficient amount of the essential amino acids required by the other strain, they would successively grow and thereby establish mutualism.

We measured the supply of amino acids from each strain in monoculture to test whether the quantities were sufficient for the successive growth of both strains in coculture. Figs. 1B and 1C show the supply of Leu from I^–^ cells in monoculture, with and without the addition of 10 µM Ile, respectively, and Figs. 1D and 1E show the supply of Ile from L^–^ cells in monoculture, with and without the addition of 10 µM Leu, respectively. Before inoculation of these cultures, we washed each strain with minimal media not containing amino acids to exclude the carry-over of supplements from preculture (see Methods). Obviously, both strains did not grow without the addition of amino acids (Fig. 1C and 1E). We measured the concentrations of Leu and Ile in the culture media using a bioassay (see Methods), and expressed these as the cell concentration of L^–^ and I^–^ cells which can be produced by the amount of amino acid supplied, respectively. In every case (Fig. 1B–E), the final concentrations of an amino acid in the recipient cell were always less than the maximum concentrations of the donor cell. That is, the nutrient supply from the donor cells was insufficient to produce an equal amount of nutrients in the recipient cells, and was therefore insufficient to sustain the net growth of both strains [20]. These results implied that any adaptation to the mutualism, such as an increase in the nutrient supply, needs to occur in coculture for the successive growth of both strains.

Despite the insufficient amino acid supply in monoculture, both strains grew to saturation (around 10^8^ to 10^9^ cells/ml) in coculture (Fig. 1F). Initially, I^–^ cells grew (red • at <10 h), followed by L^–^ cells (green ▴ at ∼20 h). Qualitatively, the initial growth of I^–^ cells was consistent with the results of amino acid supplementation in monoculture as follows. In monoculture, I^–^ cells supplied Leu only after growth and the uptake of Ile (Fig. 1B and 1C), while L^–^ cells supplied Ile regardless of growth (Fig. 1D and 1E). These results suggested that initially L^–^ cells supplied Ile promoting the growth of I^–^ cells. In addition to the initial growth of I^–^ cells, the amount of Leu was detected at time 0 in coculture, as shown in Fig. 1F (blue +). As I^–^ cells supplied Leu only when they consumed Ile, these results indicated that I^–^ cells consumed Ile supplied from L^–^ cells and then supplied Leu just after mixing but prior to sampling. However, quantitatively, the initial growth of I^–^ cells was inconsistent with the results from monoculture. I^–^ cells grew to greater than twice the concentration of L^–^ cells (Fig. 1F, red • at <10 h). That is, the Ile supply from L^–^ cells was sufficiently high to produce a greater concentration of I^–^ cells than L^–^ cells, which was different from the results of monoculture described above (Fig. 1D and 1E). The final concentration of Ile was also significantly higher than that of L^–^ cells in coculture (Fig. 1F, orange ×). The inconsistency in the quantity of Ile supplied by L^–^ cells in coculture and monoculture suggested the enhanced supply of Ile from L^–^ cells on encountering I^–^ cells. It should be noted that L^–^ cells did not show significant growth before nine hours in coculture when the Ile supply from L^–^ cells already appeared to be enhanced (Fig. 1F), which indicates that enhancement of the Ile supply from L^–^ cells did not require the population growth of strain L^–^ itself. Also, enhancement of the Leu supply from I^–^ cells was detected in coculture (Fig. 1F), as discussed later.

**Figure 2:**
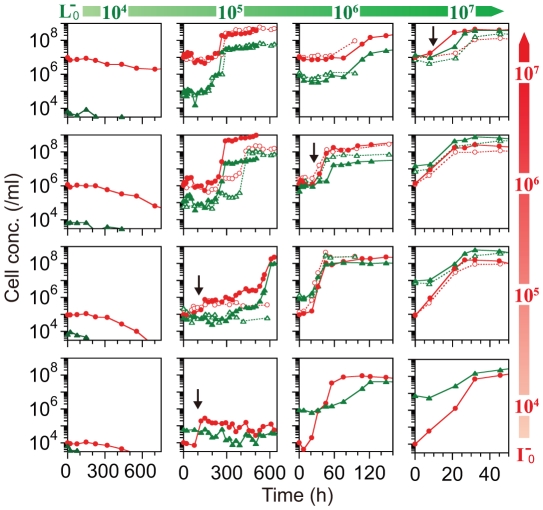
Coculture growth at various initial concentrations of I^–^ and L^–^ cells (I^–^_0_ and L^–^_0_, respectively). I^–^ and L^–^ concentrations are shown as red circles and green triangles, respectively. Two representative sets of data are shown as closed and open symbols, except when either I^–^_0_ or L^–^_0_ was 10^4^/ml, then only one set of data was obtained. When I^–^_0_ and L^–^_0_ were both at a concentration of 10^5^/ml, mutualism had been established six out of nine times. The features of the mutualism, discussed in the text, were reproducible, although the time scale varied about two-fold. The initial increase in the concentration of I^–^ cells to approximately 10-fold greater than that of L^–^ cells after the lag phase is depicted by arrows.

We investigated the growth kinetics of the cocultures at various initial cell concentrations of strains I^–^ and L^–^ (Fig. 2). The cells entered stationary phase at around 20–30, 40–120, and 300–600 h when the initial cell concentration of strain L^–^ was 10^7^, 10^6^ and 10^5^/ml, respectively. Cell growth was not observed when the initial cell concentration of strain L^–^ was 10^4^/ml. On the other hand, clear dependency on the initial cell concentration of strain I^–^ was not observed. The difference in the dependencies on the initial concentration of I^–^ and L^–^ cells was consistent with the differences in the features of nutrient supply found in the monocultures: only L^–^ cells supplied Ile even in the absence of amino acids in monoculture as described above (Fig. 1E). These results suggested that L^–^ cells initiated the first steps towards establishing mutualism in coculture.

The time courses of the cocultures also showed another feature. In some cases, the initial growth of I^–^ cells reached a concentration of up to approximately 10-fold greater than that of L^–^ cells (Fig. 2, depicted by arrows). This suggested that the Ile supply from L^–^ cells was sufficiently high to produce this concentration of I^–^ cells. As the Ile supply from L^–^ cells was insufficient to produce an I^–^ cell concentration equal to that in monoculture, which was equivalent to the initial lag time in coculture, the sufficient supply of Ile from L^–^ cells in coculture suggested that L^–^ cells changed to a "high supplier" phenotype prior to growth. Indeed, a mathematical model assuming the change in L^–^ cell phenotype could explain the time course of coculture (Fig. S1). It should also be noted that the lag period was dependent on the initial cell concentration of L^–^ cells in the coculture, which suggested that the interactions between cells were required for the change in L^–^ cell phenotype.

**Figure 3:**
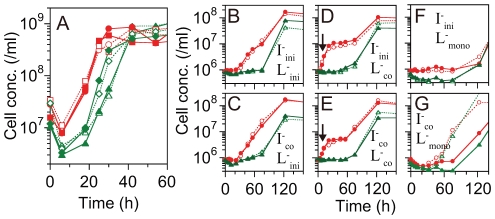
Population dynamics of the membrane coculture and re-coculture. (A) Identical dynamics between the membrane coculture (red circle for I^–^ and green triangle for L^–^) and the coculture without the membrane (red square for I^–^ and green diamond for L^–^). For the membrane coculture, I^–^ and L^–^ cells were inoculated into minimal media separated by a membrane, which was permeable to amino acids but not to *E. coli*. (B–G) The time course of the cell concentration in the re-coculture. For all re-cocultures, each sample of *E. coli* from mid-membrane culturing was washed with minimal media lacking amino acid supplements, and then mixed at an initial concentration of 10^6^/ml for both strains. The conditions for I^–^ and L^–^ cells used for each re-coculture is indicated on the graphs, where the subscripts “ini,” “co,” and “mono” refer to 0 h of membrane culture, 23 h of membrane coculture, and 23 h of membrane monoculture, respectively (see the text for details). An initial increase in the cell concentration of I^–^ cells without the lag phase was observed only in the coculture containing L^–^_co_ cells (depicted by arrows). Closed and open symbols represent replicates of two individual cultures.

To directly observe the change in L^–^ cells to a high supplier phenotype, we tested reconstituted cocultures (re-coculture) using L^–^ cells prepared from mid- coculture (Fig. 3). To prepare L^–^ cells separately from I^–^ cells from mid-coculture, we inoculated each strain into media separated by a membrane, which was permeable to amino acids but not to *E. coli* (membrane coculture) (see Methods). The time course of the membrane coculture was almost the same as that of coculture without membrane separation (Fig. 3A). For the re-coculture, we used both strains harvested from three different culture conditions: (i) at the log phase in monoculture with the addition of the required amino acids, which is the same as the initial state (0 h) of membrane coculture (I^–^_ini_ and L^–^_ini_), (ii) at 23 h of membrane coculture, when I^–^ cells had grown to a concentration approximately 10-fold higher than that of the L^–^ cells (Fig. 3A) (I^–^_co_ and L^–^_co_), and (iii) at 23 h of membrane monoculture in the absence of amino acids, when both strains were not growing (I^–^_mono_ and L^–^_mono_). Before inoculation of the re-cocultures, we washed each strain with minimal media to exclude supplements carried over from the first membrane cultures. Fig. 3B–E show the time courses of the re-cocultures comprising I^–^_ini_ and L^–^_ini_, I^–^_co_ and L^–^_ini_, I^–^_ini_ and L^–^_co_, and I^–^_co_ and L^–^_co_ cells, respectively. Only the re-coculture containing L^–^_co_ cells showed initial growth of I^–^ cells without a lag phase (Fig. 3D and 3E, arrows). These results indicated that L^–^_co_ cells were high suppliers of Ile at time 0 in the re-coculture, in contrast to L^–^_ini_ cells. It is worth noting that L^–^_ini_ cells represent the initial state of L^–^_co_ cells in the first membrane coculture, *i.e.*, L^–^ cells change to a high supplier phenotype in the first membrane coculture. Re-coculture containing L^–^_mono_ cells exhibited a lag phase before the initial growth of I^–^ cells (Fig. 3F and 3G). These results indicated that L^–^_mono_ cells were not high suppliers, like L^–^_co_ cells, and the change to a high supplier phenotype was dependent on coculturing. A significant change in I^–^ cells was not detected (Fig. 3B and 3C) in the re-coculture, although the oversupply of Leu from strain I^–^ was observed in Fig. 1F. These results experimentally confirmed that L^–^ cells changed to a high supplier phenotype in coculture prior to their own growth.

**Figure 4:**
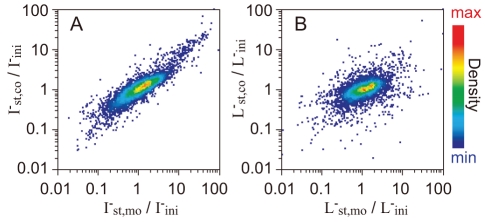
The change in gene expression of cells in coculture compared with cells in monoculture. The ratios of the intensity of expression of each gene at the stationary phase of coculture (subscript “st,co”; see text for the details) and monoculture (“st,mo”), compared with those in their common initial state (“ini”), were calculated for all 4345 genes and plotted on the vertical and horizontal axes, respectively. The horizontal axis represents the changes from the log phase in the monoculture to the stationary phase in the monoculture, *i.e.*, only changes in the phase of growth. The vertical axis represents the changes from the log phase in the monoculture to the stationary phase in the coculture, *i.e.*, sum of the coculture-specific changes and the changes in the phase of growth. The dot densities are shown as a heat map. (A) Changes in gene expression of I^–^ cells. The slope of the linear regression of the log value was 0.67 and the correlation coefficient (*R*) was 0.89. (B) Changes in gene expression of L^–^ cells. The slope of the linear regression of the log value was 0.37 and *R* was 0.54.

These findings raised the question: how does gene expression change in the two strains during coculture? To investigate this, we carried out a comprehensive analysis of gene expression in these two strains. To harvest each strain separately from coculture, we again employed membrane coculture. Using a DNA microarray, we measured and compared the expression intensities of all 4345 genes of each strain cultured under three different conditions. These three conditions were: (i) at the log phase in monoculture, which is the same as the initial state (0 h) of membrane coculture, as described above (I^–^_ini_ and L^–^_ini_), (ii) at the stationary phase (45 h) of membrane coculture (I^–^_st,co_ and L^–^_st,co_), and (iii) at the stationary phase (45 h) of monoculture after growth in the presence of the required amino acids (I^–^_st,mo_ and L^–^_st,mo_). As *E. coli* is known to substantially change its gene expression depending on the growth phase [23], samples were taken at 45 h (not 23 h) to identify coculture-specific changes by comparing samples at the same phase (stationary phase). In the I^–^ strain, the changes in gene expression from I^–^_ini_ to I^–^_st,co_ strongly correlated with those from I^–^_ini_ to I^–^_st,mo_ (Fig. 4A), that is, the dominant changes were dependent on the growth phase. This correlation was also observed in L^–^ cells and the slope of linear regression was smaller than of I^–^ cells (Fig. 4B), which may have been because the time after entering stationary phase was shorter in L^–^_st,co_ cells than in I^–^_st,co_ cells (Fig. 3A). More importantly, in strain L^–^, the correlation coefficient was smaller than that in strain I^–^ (Fig. 4B). These results indicated that the change in gene expression of L^–^ cells in coculture was more coculture-specific than that of I^–^ cells.

The next question raised was: which categories of genes were involved in the coculture-specific changes in expression in L^–^ cells shown in Fig. 4B? Initially, we focused on genes that showed significantly induced or repressed expression in coculture compared to monoculture, *i.e.*, those in which the ratio of gene expression in L^–^_st,co_ cells to that in L^–^_st,mo_ cells was greater than three or lower than one-third. We statistically screened the up-regulated and down-regulated gene categories to which the significantly induced or repressed genes belonged (Table 1). For the gene categories, we adopted the “cellular processes” category in the Gene Ontology (GO) database [24], and the categories for genes regulated by sigma factors in the database, RegulonDB [25]. In the "cellular processes" category of the GO database, 14 categories out of 124 were found to be up-regulated, and most of these up-regulated categories were related to anabolism, such as the biosynthesis of amino acids (tryptophan, proline, methionine, phenylalanine, leucine, cysteine, and chorismate, which is a precursor of tyrosine, phenylalanine and tryptophan), polyamines and proteins. In contrast, of the nine down-regulated categories, most were related to catabolism, such as the various energy cycles (glyoxylate and tricarboxylic acid cycles), fatty acid oxidation, and the catabolism of amino acids, aminobutyrate and carbohydrates. Some of these up- and down-regulated categories were also identified when comparing L^–^_st,co_ and L^–^_ini_ cells (Table 1, indicated by arrows). Although L^–^ cells oversupplied Ile in coculture, no significant increase was found in the expression of genes related to Ile biosynthesis or Ile transport in L^–^_st,co_ cells compared with both L^–^_st,mo_ and L^–^_ini_ cells (Fig. S2). It is worth noting that the results of liquid chromatography showed that the predominant supplement from L^–^ cells required by I^–^ cells was Ile (Fig. S3). Among the genes regulated by sigma factors, we detected the down-regulation of genes regulated by Sigma 70, the housekeeping sigma factor [26], during the change from L^–^_ini_ to L^–^_st,co_ cells (arrows, Table 1) and the change from L^–^_ini_ to L^–^_st,mo_ cells. These results were consistent with the change in growth phase to stationary phase. Although the down-regulation of Sigma 70 genes occurred in both L^–^_st,co_ and L^–^_st,mo_ cells, the gene expression significantly differed between L^–^_st,co_ and L^–^_st,mo_ cells (Fig. 4B). Therefore both up- and down-regulation was found when comparing L^–^_st,co_ and L^–^_st,mo_ cells. Down-regulation of the glutamine biosynthesis gene category correlated with the down-regulation by Sigma 54, the sigma factor controlling nitrogen usage [26], [27]. As glutamine biosynthesis opposes glutamate biosynthesis leading to the biosynthesis of other amino acids, the down-regulation of glutamine biosynthesis is not inconsistent with the up-regulation of the anabolic categories. As above, we found that the coculture-specific changes in gene expression in L^–^ cells were not related to the local activation of the biosynthesis and transport of isoleucine, but were related to the global activation of anabolic metabolism.

**Table 1:** The categories of up-regulated and down-regulated genes in L^–^_st,co_ cells.

Database	Up-regulated categories	Down-regulated categories
GO	0000162 tryptophan biosynthesis ↑	0006071 glycerol metabolism
Cellular	0000270 peptidoglycan metabolism	0006097 glyoxylate cycle ↓
processes	0006310 DNA recombination	0006099 tricarboxylic acid cycle ↓
	0006412 protein biosynthesis	0006542 glutamine biosynthesis ↓
	0006561 proline biosynthesis	0009063 amino acid catabolism ↓
	0006596 polyamine biosynthesis	0009450 aminobutyrate catabolism
	0006790 sulfur metabolism	0016052 carbohydrate catabolism ↓
	0009086 methionine biosynthesis ↑	0019395 fatty acid oxidation
	0009094 L-phenylalanine biosynthesis	0042594 response to starvation ↑
	0009098 leucine biosynthesis (except *leuB*) ↑	
	0009243 O antigen biosynthesis	
	0009257 10-formyltetrahydrofolate biosynthesis	
	0009423 chorismate biosynthesis ↑	
	0019344 cysteine biosynthesis	
Sigma	Sigma 70 ↓	Sigma 54 ↓
factors		Sigma 70 ↓

The categories were screened by comparing between L^–^_st,co_ and L^–^_st,mo_ cells as coculture-specific changes. In the screened categories, upward and downward arrows (↑ or ↓) at the end of the category name represent up-regulated or down-regulated categories, respectively, in L^–^_st,co_ cells relative to L^–^_ini_ cells.

## Discussion

In our synthetic model of obligate mutualism comprising two auxotrophs of *E. coli*, strains I^–^ and L^–^, the increase in the Ile supply from L^–^ cells occurred before the population growth of L^–^ cells, and both strains grew successively thereafter in coculture. We found that the increase in the Ile supply from L^–^ cells depended on coculture with I^–^ cells and was accompanied by coculture-specific changes in the gene expression of L^–^ cells. This change in L^–^ cells in coculture was not related to the local activation of the biosynthesis and transport of isoleucine, but was related to the global activation of anabolic metabolism.

What is the mechanism behind the phenotypic change in L^–^ cells to become “high suppliers” of isoleucine? There are two possibilities: (i) a fraction of high suppliers preexisted in the initial population and their fraction in the L^–^ population increased in the coculture (natural selection), (ii) L^–^ cells changed their phenotypes in response to the changes in the environment from monoculture to coculture (phenotypic plasticity). We can rule out neither possibility completely.

Let us first assume that (i) is true, and estimate an approximate range of the fraction of preexisting high suppliers in the initial population (*f*_H0_). At first, the supply of Ile from high suppliers was about 10-fold higher than that of “normal” L^–^ cells. Therefore, *f*_H0_ would be less than 10%, otherwise the supply of Ile from L^–^ cells at the initial state or in the monoculture would be higher than the experimental results. Second, the shortest time until the initial growth of I^–^ cells was about 10 hours. By then the high suppliers had already been the majority in the L^–^ population and their concentration was approximately 10^7^/ml (Fig. 1F). For the concentration of the high suppliers to become 10^7^/ml in 10 hours from the initial concentration 10^7^·*f*_H0_/ml, *f*_H0_> exp(–10*g*_H_) must be satisfied, where *g*_H_ is the growth rate of the high suppliers. Even if only high suppliers grew at a maximum growth rate of L^–^ cells (*g*_H_ = 0.4/h, Table S1), *f*_H0_>2% was required. *f*_H0_ thus can be estimated as 2%<*f*_H0_<10%. Note that there is no reason why only high suppliers grew at a maximum rate in the environment, where the normal L^–^ cells did not grow. In the mixed liquid culture, all of the L^–^ cells were considered to acquire Leu from the media (not from I^–^ cells directly) in a homologous environment (actually, physical contacts were negligible; Fig. 3A). Moreover, *f*_H0_ must be kept in this range in monoculture because the time until the initial growth of I^–^ cells were reproducible even when we used another clone of L^–^ cell for the preparation of the initial population.

We then discuss about the possibility (ii). *E. coli* is known to alter its phenotype in response to environmental changes, such as amino acid starvation, and this is known as a stringent response [28] and represents a kind of phenotypic plasticity. As L^–^ cells were subject to Leu starvation at the initial in coculture, they would have changed their phenotype as the stringent response. This response might have been preserved even after their growth in which they had already been released from Leu starvation. Indeed, the up-regulation of amino acid synthesis, which is known to occur in the stringent response [28], was observed in L^–^_st,co_ relative to L^–^_st,mo_ (Table 1). However, although L^–^ cells in monoculture without Leu (L^–^_mono_) were subject to Leu starvation, they did not change to the high supplier phenotype (Fig. 3F and 3G). In our experiments, L^–^ cells changed to a high supplier phenotype only in coculture, and the genes related to Ile biosynthesis and transport were not significantly induced in these cells (Fig. S2), in contrast, these genes are induced during the stringent response [28]. It is known that Ile uptake is increased and amino acid permeability is decreased during the stringent response [28], which seems to oppose the extracellular leakage of Ile. Our results might therefore indicate that the phenotypic change in L^–^ cells was related not only to the known stringent responses, but also to other responses due to the interaction among strains via the media. As both the I^–^ and L^–^ strains were constructed by a single gene deletion from the same original strain, DH1 (see Methods), the substances supplied by them via cell leakage would be expected to be almost the same. Thus, the interaction between these strains is unlikely due to the expression of a specific substance, as is the case in quorum sensing [29], but is more likely due to a global change of the composition of multiple substances [30], [31]. This might be consistent with the observed global activation of expression of genes involved in anabolic metabolism (Table 1). It is worth noting that a similar phenomenon was observed in a synthetic mutualism comprising I^–^ cells and an uracil auxotroph (Fig. S4), therefore, the change in L^–^ cell phenotype is not due to the similarities between the metabolism of Ile and Leu. Further studies are required to fully elucidate the mechanism behind the phenotypic change in strain L^–^.

It is unknown to what extent such an adaptation to a first encounter contributes to the establishment of a nascent mutualism in nature. However, this potential would provide insight into the positive factors required for the establishment of natural mutualisms. We do not believe that an adaptation, such as that described in this study, results in the establishment of nascent mutualism in every case, because we actually failed to establish mutualism with some combinations of auxotrophs (such as a glutamine auxotroph and an uracil auxotroph). Synthetic mutualism also failed to be established in other types of organisms without the introduction of metabolite-overproducing mutations [9], [20]. However, due to the great variety of organisms in nature [3], [32], organisms inevitably encounter other kinds of organisms and have the opportunity to establish a nascent mutualism, where such an adaptation to this first encounter can facilitate this process. It is worth noting that such an adaptation to first encounter might be a kind of the phenotypic plasticity in response to a new environment [33], [34], [35], [36], [37], [38], [39], [40], and the adaptation in response to a first encounter might have evolved because the organisms possessing this potential should survive in the bio-diversified nature. Field studies are required to fully investigate the contribution of such an adaptation to the establishment of nascent mutualism in nature.

The simplicity of the synthetic model of mutualism used in this study enabled us to identify unexpected and quantitative changes in the organisms. Experimental ecosystems not only provide empirical proof of theories but also highlight unexpected phenomena, such as the unknown potential of organisms which may lead to novel theories. For example, in another bacterial system, Fiegna *et al*. found that a single point mutation changed a cheater into a cooperator with a tolerance to exploitation by the cheater [41], [42]. Without the simplicity of the system, it would have been impossible to detect such a phenomenon. For future studies, synthetically-constructed experimental ecosystems combining naturally non-interacting species [21], [43], [44], [45], [46] and reconstructing interactions using genetic modifications [16], [19], [20], [47], [48], [49], [50], [51], [52], [53], would be invaluable for the detection of other unexpected phenomena.

The simplicity of our synthetic model of mutualism will enable further studies to experimentally resolve some of the remaining questions, such as the molecular mechanisms behind the observed adaptation in L^–^ cells and the evolutionary pathway of this mutualism. Our findings may also contribute to the study of mutualism in other organisms, including higher organisms, and in field studies investigating natural ecosystems.

## Materials and Methods

### Construction of *E. coli* strains

The *E. coli* strain DH1Δ*ilvE*::(*dsred.T3*-*cat*), designated I^–^, was constructed from the *E. coli* strain DH1 (National BioResource Project, National Institute of Genetics, Shizuoka, Japan), by replacing the chromosomal *ilvE* gene with a foreign DNA fragment, *P_tetA_-dsred.T3-P_cat_-cat*, comprised of a reporter gene (*dsred.T3*) and the chloramphenicol resistance gene (*cat*). The *dsred.T3* gene and the *cat* gene were transcribed from their promoters in opposite directions. The DNA fragment was amplified by PCR using the template, pPROTetE.333-tetT3, in which the *dsred.T3* fragment (a gift from Dr. B.S.Glick, The University of Chicago [54]) along with the PtetA fragment with *Xho*I and *Not*I recognition sites, had been subcloned into the corresponding site of the plasmid, pPROTet.E333-lacZ (Clontech), using the following primers, reccatilvEr (5′-AACAAATCCGCGCCTGAGCGCAAAAGGAATATAAAATTACGCCCCGCCCTGCCACT-3′) and ilvE-T3cat-r (5′-TAAATGGGACGGTGCGTGCCGTCCCATTTTTTGTATATTATCACAGGAACAGGTGG-3′). Homologous recombination was performed as described previously [55], [56]. The *E. coli* strain DH1Δ*leuB*::(*gfpuv5-Km^r^*), designated L^–^, was constructed from the *E. coli* strain DH1, by replacing the chromosomal *leuB* gene with a foreign DNA fragment, *P_tetA_-gfpuv5-P_Km_-Km^r^*, comprising a reporter gene (*gfpuv5*) and the kanamycin resistance gene (*Km^r^*). The DNA fragment was amplified by PCR using the template, pGAG-2 [57] and primers, leuB-kanIG-f (5′-GCTCAACACAACGAAAACAACAAGGAAACCGTGTGATTAGAAAAACTCATCGAGCA-3′) and leuB-IGkan-r (5′- CGTCGAACAATTTTTCGTATAACGTCTTAGCCATGAATTATCATTTGTAGAGCTCA-3′).

### Culture conditions

All cultures were grown at 37°C in well-mixed minimal media modified with M63 (pH 7.0, 62 mM K_2_HPO_4_, 39 mM KH_2_PO_4_, 15 mM ammonium sulfate, 1.8 µM FeSO_4_-7H_2_O, 15 µM thiamine hydrochloride, 0.2 mM MgSO_4_-7H_2_O and 22 mM glucose; mM63 [56]). Amino acids were added to the media when appropriate. Before culturing, we washed *E. coli* strains with the minimal media without amino acids to exclude the carry-over of supplements from preculture. For the membrane culture, we used cell culture inserts with a pore size of 0.45 µm at a density of 10^8^/cm^2^, and used six-well cell culture companion plates for the inserts (BD Falcon, Franklin Lakes, NJ, USA). The initial concentration of I^–^ and L^–^ cells are depicted at time 0 in the figures or described in the figure legends.

### Measurement of cell concentrations

We measured the cell concentration relative to a known concentration of fluorescent beads (Fluoresbrite YG Microspheres, 3 µm; Polysciences Inc., Warrington, PA, USA) using a Cytomics TM FC500 Flow Cytometer (Beckman Coulter, Inc, CA, USA) by loading culture samples mixed with the beads. A 488 nm argon excitation laser was employed and band-pass filters of 515–535 and 610–630 nm were used to measure green and red fluorescence, respectively. Clusters of red and green cells, and the fluorescent beads, were clearly segregated (Fig. S5), and each cell concentration was calculated from these counts.

### Measurement of amino acid concentrations using a bioassay

To measure the Ile concentration of a culture, the culture was passed through a 0.2 µm filter and the supernatant was supplemented with a one-sixth volume of the mM63 media and inoculated with I^–^ cells at 10^4^/ml. Then the Ile concentration was obtained by multiplying the saturation concentration of I^–^ cells (>48 h) by six. The Leu concentration of a culture was obtained using the same method for strain L^–^. It is worth noting that when Ile was added to the monoculture of I^–^ cells in mM63 media, the concentration of added Ile and the saturation concentration of I^–^ cells was proportional, with a constant of 9.8×10^5^ (cells/ml)/(µM). The same was also true for the added Leu concentration and the saturation concentration of L^–^ cells, with a constant of 1.8×10^6^ (cells/ml)/(µM) (both correlation coefficients: *R*>0.98).

### Gene expression analysis

*E. coli* gene expression was examined using a GeneChip® *E. coli* Genome Antisense Genome Array according to the Expression Analysis Technical Manual (Affymetrix, 2004). The expression analyses of co-culture samples were performed with two technical replicates using two different target cDNAs separately prepared for each sample. The expression level of each gene was computed according to the FH model [58]. The estimated expression levels were normalized using a quantile normalization method [59]. For the analysis of the gene categories, we used three as the threshold for the ratio of gene expression to determine whether the expression of a gene had changed. When we calculated the ratio of gene expression for each of the 4345 genes between the two replicates of L^–^_st,co_ cells in individual cocultures, the ratios were less than three for 98% of genes. To screen the categories that were significantly up- or down-regulated, we used a one-side binomial test at the significance level of 0.01.

## Supporting Information

Figure S1 Mathematical model of the growth kinetics of the coculture. (A) Mathematical model of the growth kinetics. [X] indicates the concentration in the culture media of X. We defined active cells of strains I^−^ and L^−^ as I^−^_act_ and L^−^_act_, respectively, for the following reason. When we measured the cell concentration as colony forming units (cfu) under starvation conditions, the concentration determined by cfu was less than the concentration determined by analysis of fluorescent particles by flow cytometry (Fig. S5B and S5C). Although it was difficult to determine whether cells were alive or dead, we defined a cell being able to form a single colony as an active cell. This model neglects the decrease in [I^−^] and [L^−^] because it was slow (Fig. S5B and S5C). The symbol S represents glucose as a carbon source in the minimal media, which only determines the saturation concentration (we set 10^9^/ml for the simulation in Fig. S1B). The explanations and the values of the parameters are shown in Table S1. This model is based on the Monod model with the maintenance rate [60]. The specific character of this model is the heterogeneity of the supply function of the amino acid between I^−^ and L^−^ cells, as experimentally shown in Fig. 1. A mathematical model assuming these two types of nutrient supply has been reported for another obligate mutualism comprising two bacteria isolated from soil microcosms [61]. As I^−^ cells supplied Leu only after growth, *a_L_* was defined as the number of L^−^ cells produced in the presence of Leu from a single new I^−^ cell until its death in the culture. In this model, a mathematically solved necessary condition for the stable growth of both strains is *a_L_k_L_*/*m_L_*>1. *k_L_*/*m_L_* represents the number of I^−^ cells produced in the presence of Ile from a single new L^−^ cell until its death in the culture. In our experiments, *k_L_*/*m_L_* was less than one in monoculture (Fig. 1D and 1E) and at the lag phase in coculture (Fig. 2 and 3), but was nearly 10 after the lag phase in coculture (Fig. 2 and 3). (B) Comparison between the simulation results of the model and the experimental results shown in Fig. 2. The parameters used for the simulation (Table S1) assumed the cooperative change of L^−^ cells to be *k_L_* = 0.4/h, which was determined from the experimental results of coculture (Fig. 3D). When *k_L_* = 0.007/h, which is the value determined from the experimental results of monoculture (Fig. 1E), both strains must not grow successively because *a_L_k_L_*/*m_L_*<1. As the model neglects the lag time until the change in L^−^ cells, the deviations of the simulation results from the experimental results for the initial growth of I^−^ cells are shown. (C) Comparison between the simulation results of the model and the experimental results shown in Fig. 1F. The simulation also roughly fit to the experimental results regarding the amino acid concentrations.
(EPS)

Figure S2 The change of the expression of genes related to Ile biosynthesis and transport in L^−^ cells. The black and gray bars show the ratio of the expression of each gene in L^−^_st,co_ and L^−^_st,mo_ cells compared to that in L^−^_ini_ cells, respectively, where L^−^_st,co_, L^−^_st,mo_ and L^−^_ini_ represent the state of L^−^ cells at the stationary phase of coculture, the stationary phase of monoculture and the growth phase as their common initial state, respectively (see text for details). Genes related to Ile biosynthesis and transport are depicted as “Biosynthesis” and “Transport,” respectively, under their gene names. None of the Ile biosynthesis-related genes were induced in L^−^_st,co_ cells compared with L^−^_ini_ cells or in L^−^_st,co_ cells compared with L^−^_st,mo_ cells, using three-fold (red line) as the significant threshold (see Methods). None of the Ile transport-related genes were significantly changed between any two of the three conditions.
(EPS)

Figure S3 The ratio of the concentration of amino acids determined by HPLC to those determined by a bioassay. We determined the quantity of Ile and Leu in the supernatants of the cultures in which each strain reached saturation phase in the presence of the required amino acid (1 µM Ile or 1 µM Leu for I^−^ or L^−^ cells, respectively) using two different methods: HPLC and a bioassay. The results indicated that the supplied nutrient from L^−^ cells that compensated for the Ile auxotrophy of I^−^ cells consisted mainly of Ile, while the supplied nutrient from I^−^ cells that compensated for the Leu auxotrophy of L^−^ cells consisted mainly of substances other than Leu. Methods for the bioassay are described in the text and the methods for HPLC are described below. We added Ile and Leu to the supernatants (both 0.05 µM) to raise the concentrations in the supernatants above the detection range of HPLC. As an internal standard, norleucine was also added to 0.25 µM in the supernatant. The resultant solutions were derivatized by phenylisothiocyanate (Wako, Osaka, Japan) and applied to a reverse phase HPLC on a Waters LC Module 1 (Waters Corporation, MA, USA) with a column of Wakosil-PTC (4.0×250 mm, Wako, Osaka, Japan). The column was soaked in a circulating water bath at 40°C. The mobile phase comprised 60 mM sodium acetate (pH 6.0) and acetonitrile (94∶6) as eluant A; eluant B consisted of 60 mM sodium acetate (pH 6.0) and acetonitrile (40∶60). Gradient elution was employed according to the following linear program: time 0, 0% eluant B; 20 min, 70% eluant B; 21 min, 100% eluent B. The flow rate was 1 ml/min. Amino acid derivatives were detected by their absorbance at 254 nm.
(EPS)

Figure S4 Basic design and cell growth of the synthetic mutualism comprising I^−^ cells and a Ura auxotroph (U^−^). (A) Schematic diagram of the synthetic mutualism. Two auxotrophs of *E. coli*, strains I^−^ and U^−^, supply nutrients to each other to form a potential mutualism. (B–E) Cell growth and nutrient release properties of the monocultures. The concentration of Ura was determined by a bioassay, as was the concentration of Ile and Leu. The concentration of Ura or Ile is indicated as the density of U^−^ or I^−^ cells which can be produced by that amount of Ura or Ile, respectively. When the nutrient concentration was not detected (under the detection limit 10^5^/ml), we plotted it at 10^5^/ml. (B and C) The time courses of the concentration of Ura (blue square) and I^−^ cells (red circle) in monoculture. (B) 10^5^/ml of I^−^ cells were inoculated into minimal media along with 10 µM of Ile. (C) 10^7^/ml of I^−^ cells were inoculated into minimal media along with Ile. (D and E) The time courses of the concentration of Ile (orange square) and U^−^ cells (green circle). (D) 10^5^/ml of U^−^ cells were inoculated into minimal media along with 10 µM of Ura. (E) 10^7^/ml of U^−^ cells were inoculated into minimal media without the addition of Ura. (F) The time courses of the concentration of I^−^ cells (red symbols) and U^−^ cells (green symbols) in coculture. 10^7^/ml of both I^−^ and U^−^ cells (• and ▴, respectively), 10^7^/ml of I^−^ and 10^6^/ml of U^−^ (○ and Δ), or 10^6^/ml of I^−^ and 10^7^/ml of U^−^ (× and +) were inoculated into minimal media in the presence of Ile and Ura. These results were similar to the results of the mutualism with I^−^ and L^−^ cells shown in Fig. 1. The final concentrations of nutrients were always less than the maximum concentrations of the donor cell in monoculture (B–E), which meant that the nutrient supplies from these strains in monoculture were insufficient for the continuous growth of both strains in coculture. Despite the insufficient level of nutrient supply in monoculture, both strains grew to saturation in coculture with all of the initial cell concentrations used (F). Strain U^−^ (DH1Δ*leuB*::(*gfpuv5-Km^r^*)) was constructed from DH1 cells, as was strain L^−^, by replacing the chromosomal *pyrE* gene with a foreign DNA fragment comprising a reporter gene (*gfpuv5*) and the kanamycin resistance gene (*Km^r^*).
(EPS)

Figure S5 Measurement of the cell concentrations by flow cytometry (FCM). (A) The dot plot of the data collected from the coculture by FCM. I^−^ cells (red dots), L^−^ cells (green dots), and the calibration beads (yellow dots) were clearly segregated. The definitions of these three particles were as follows: *x*>1.6 (red dashed line), *y*>15 (red dotted line), and Log_10_ *y*>Log_10_ *x*+0.43 (red solid line) for I^−^ cells, where *x* and *y* represent green and red florescent intensities (a. u.), respectively; *x*>15 (green dashed line) and *y*<0.036*x*+10 (green solid line) for L^−^ cells; *x*>540 (yellow dashed line), *y*>0.036*x*+10 (green solid line), and Log_10_ *y*<0.256 (Log_10_ *x*)^2^ (yellow solid line) for the beads. (B and C) The difference in the cell concentrations determined by FCM (closed symbols) and colony forming units (cfu) (open symbols) for I^−^ (B) and L^−^ cells (C). 10^7^/ml (black circles) or 10^5^/ml (blue squares) of the cells were inoculated into minimal media without the addition of any amino acid, or 10^5^/ml of the cells were inoculated into minimal media with 10 µM of the required amino acid (Ile for I^−^ cells and Leu for L^−^ cells) (red triangles). Although there was little difference between the concentration determined by FCM and the concentration determined by cfu at time 0, the concentration determined by cfu decreased more quickly than the concentration determined by FCM. Therefore, although it is difficult to determine whether a cell is alive or dead, we defined an active cell as a cell that was able to form a single colony in the mathematical model in Fig. S1A.
(EPS)

Table S1 Explanations and values of the parameters used in the mathematical model.
(DOC)

## References

[1] Herre EA, Knowlton N, Mueller UG, Rehner SA (1999). The evolution of mutualisms: exploring the paths between conflict and cooperation.. Trends Ecol Evol.

[2] Sachs JL, Simms EL (2006). Pathways to mutualism breakdown.. Trends Ecol Evol.

[3] McArthur JV (2006). Microbial ecology: an evolutionary approach..

[4] Douglas AE (1994). Symbiotic interactions..

[5] Boucher DH, James S, Keeler KH (1982). The Ecology of Mutualism.. Annu Rev Ecol Syst.

[6] Begon M, Harper JL, Townsend CR (1996). Ecology: individuals, populations, and communities..

[7] Aanen DK, Eggleton P, Rouland-Lefevre C, Guldberg-Froslev T, Rosendahl S (2002). The evolution of fungus-growing termites and their mutualistic fungal symbionts.. Proc Natl Acad Sci U S A.

[8] Machado CA, Robbins N, Gilbert MT, Herre EA (2005). Critical review of host specificity and its coevolutionary implications in the fig/fig-wasp mutualism.. Proc Natl Acad Sci U S A.

[9] Harcombe W (2010). Novel cooperation experimentally evolved between species.. Evolution.

[10] May RM, McLean AR (2007). Theoretical ecology: principles and applications..

[11] Hamilton WD (1964). The genetical evolution of social behaviour.. I. J Theor Biol.

[12] Nowak MA (2006). Five rules for the evolution of cooperation.. Science.

[13] West SA, Griffin AS, Gardner A (2007). Evolutionary explanations for cooperation.. Curr Biol.

[14] Griffin AS, West SA, Buckling A (2004). Cooperation and competition in pathogenic bacteria.. Nature.

[15] MacLean RC, Gudelj I (2006). Resource competition and social conflict in experimental populations of yeast.. Nature.

[16] Chuang JS, Rivoire O, Leibler S (2009). Simpson's paradox in a synthetic microbial system.. Science.

[17] Rainey PB, Rainey K (2003). Evolution of cooperation and conflict in experimental bacterial populations.. Nature.

[18] Ross-Gillespie A, Gardner A, Buckling A, West SA, Griffin AS (2009). Density dependence and cooperation: theory and a test with bacteria.. Evolution.

[19] Wintermute EH, Silver PA (2010). Emergent cooperation in microbial metabolism.. Mol Syst Biol.

[20] Shou W, Ram S, Vilar JM (2007). Synthetic cooperation in engineered yeast populations.. Proc Natl Acad Sci U S A.

[21] Hillesland KL, Stahl DA (2010). Rapid evolution of stability and productivity at the origin of a microbial mutualism.. Proc Natl Acad Sci U S A.

[22] Hosoda K, Shiroguchi Y, Yamauchi Y, Kashiwagi A, Mori K (2008). Synthetic ecosystem of *Escherichia coli* for discovery of novel cooperative and self-adaptive algorithms.. Proc BIONETICS 2008: ICST.

[23] Selinger DW, Cheung KJ, Mei R, Johansson EM, Richmond CS (2000). RNA expression analysis using a 30 base pair resolution Escherichia coli genome array.. Nat Biotechnol.

[24] Riley M, Abe T, Arnaud MB, Berlyn MK, Blattner FR (2006). Escherichia coli K-12: a cooperatively developed annotation snapshot–2005.. Nucleic Acids Res.

[25] Gama-Castro S, Jimenez-Jacinto V, Peralta-Gil M, Santos-Zavaleta A, Penaloza-Spinola MI (2008). RegulonDB (version 6.0): gene regulation model of Escherichia coli K-12 beyond transcription, active (experimental) annotated promoters and Textpresso navigation.. Nucleic Acids Res.

[26] Magnusson LU, Farewell A, Nystrom T (2005). ppGpp: a global regulator in Escherichia coli.. Trends Microbiol.

[27] Reitzer L, Schneider BL (2001). Metabolic context and possible physiological themes of sigma(54)-dependent genes in Escherichia coli.. Microbiol Mol Biol Rev.

[28] Cashel M, Gentry DR, Hernandez VJ, Vinella D, Neidhardt FC, Curtiss R (1996). The stringent response.. Escherichia coli and Salmonella: cellular and molecular biology. 2nd ed.

[29] Hooshangi S, Bentley WE (2008). From unicellular properties to multicellular behavior: bacteria quorum sensing circuitry and applications.. Curr Opin Biotechnol.

[30] Huang S (2004). Back to the biology in systems biology: what can we learn from biomolecular networks?. Brief Funct Genomic Proteomic.

[31] Bar-Yam Y, Harmon D, de Bivort B (2009). Systems biology. Attractors and democratic dynamics.. Science.

[32] Reaka-Kudla ML, Wilson DE, Wilson EO (1997). Biodiversity II: understanding and protecting our biological resources..

[33] Pigliucci M (2005). Evolution of phenotypic plasticity: where are we going now?. Trends Ecol Evol.

[34] Ghalambor CK, Mckay JK, Carroll SP, Reznick DN (2007). Adaptive versus non-adaptive phenotypic plasticity and the potential for contemporary adaptation in new environments.. Funct Ecol.

[35] Waddington CH (1952). Selection of the Genetic Basis for an Acquired Character.. Nature.

[36] West-Eberhard MJ (2003). Developmental plasticity and evolution..

[37] Badyaev AV (2009). Evolutionary significance of phenotypic accommodation in novel environments: an empirical test of the Baldwin effect.. Philos Trans R Soc Lond B Biol Sci.

[38] Shimada M, Ishii Y, Shibao H (2010). Rapid adaptation: a new dimension for evolutionary perspectives in ecology.. Popul Ecol.

[39] de Jong G, Crozier RH (2003). A flexible theory of evolution.. Nature.

[40] Yamada A, Matsuyama S, Todoriki M, Kashiwagi A, Urabe I (2008). Phenotypic plasticity of Escherichia coli at initial stage of symbiosis with Dictyostelium discoideum.. Biosystems.

[41] Velicer GJ, Raddatz G, Keller H, Deiss S, Lanz C (2006). Comprehensive mutation identification in an evolved bacterial cooperator and its cheating ancestor.. Proc Natl Acad Sci U S A.

[42] Fiegna F, Yu YT, Kadam SV, Velicer GJ (2006). Evolution of an obligate social cheater to a superior cooperator.. Nature.

[43] Buchsbaum R, Buchsbaum M (1934). An Artificial Symbiosis.. Science.

[44] Tsuchiya HM, Drake JF, Jost JL, Fredrickson AG (1972). Predator-prey interactions of Dictyostelium discoideum and Escherichia coli in continuous culture.. J Bacteriol.

[45] Kim HJ, Boedicker JQ, Choi JW, Ismagilov RF (2008). Defined spatial structure stabilizes a synthetic multispecies bacterial community.. Proc Natl Acad Sci U S A.

[46] Todoriki M, Oki S, Matsuyama S, Ko-Mitamura EP, Urabe I (2002). An observation of the initial stage towards a symbiotic relationship.. Biosystems.

[47] Basu S, Gerchman Y, Collins CH, Arnold FH, Weiss R (2005). A synthetic multicellular system for programmed pattern formation.. Nature.

[48] Weber W, Daoud-El Baba M, Fussenegger M (2007). Synthetic ecosystems based on airborne inter- and intrakingdom communication.. Proc Natl Acad Sci U S A.

[49] Balagadde FK, Song H, Ozaki J, Collins CH, Barnet M (2008). A synthetic Escherichia coli predator-prey ecosystem.. Mol Syst Biol.

[50] Song H, Payne S, Gray M, You L (2009). Spatiotemporal modulation of biodiversity in a synthetic chemical-mediated ecosystem.. Nat Chem Biol.

[51] Chuang JS, Rivoire O, Leibler S (2010). Cooperation and Hamilton's rule in a simple synthetic microbial system.. Mol Syst Biol.

[52] Hu B, Du J, Zou RY, Yuan YJ (2010). An environment-sensitive synthetic microbial ecosystem.. PLoS One.

[53] Wintermute EH, Silver PA (2010). Dynamics in the mixed microbial concourse.. Genes Dev.

[54] Bevis BJ, Glick BS (2002). Rapidly maturing variants of the Discosoma red fluorescent protein (DsRed).. Nat Biotechnol.

[55] Datsenko KA, Wanner BL (2000). One-step inactivation of chromosomal genes in Escherichia coli K-12 using PCR products.. Proc Natl Acad Sci U S A.

[56] Kashiwagi A, Sakurai T, Tsuru S, Ying BW, Mori K (2009). Construction of Escherichia coli gene expression level perturbation collection.. Metab Eng.

[57] Suzuki T, Kashiwagi A, Urabe I, Yomo T (2006). Inherent characteristics of gene expression for buffering environmental changes without the corresponding transcriptional regulations.. Biophysics.

[58] Ono N, Suzuki S, Furusawa C, Agata T, Kashiwagi A (2008). An improved physico-chemical model of hybridization on high-density oligonucleotide microarrays.. Bioinformatics.

[59] Bolstad BM, Irizarry RA, Astrand M, Speed TP (2003). A comparison of normalization methods for high density oligonucleotide array data based on variance and bias.. Bioinformatics.

[60] Kovarova-Kovar K, Egli T (1998). Growth kinetics of suspended microbial cells: from single-substrate-controlled growth to mixed-substrate kinetics.. Microbiol Mol Biol Rev.

[61] Katsuyama C, Nakaoka S, Takeuchi Y, Tago K, Hayatsu M (2009). Complementary cooperation between two syntrophic bacteria in pesticide degradation.. J Theor Biol.

