# Supplementary material for: Cooperative Adaptation to Establishment of a Synthetic Bacterial Mutualism
Source: PLoS One. 2011 Feb 16;6(2):e17105. doi: 10.1371/journal.pone.0017105 (PMC3040204; doi:10.1371/journal.pone.0017105)
Supplement: Table S1 — Explanations and values of the parameters used in the mathematical model. (DOC) [file pone.0017105.s006.doc]

Table S1. Explanations and values of the parameters used in the mathematical model.

|  | Explanations | Value_mono_ | Value_co_ |
| --- | --- | --- | --- |
| *μ_I_* | Sum of the maximum specific growth rate and maintenance rate *d_I_* of I^-^ | 0.39/h * |  |
| *μ_L_* | Sum of the maximum specific growth rate and maintenance rate *d_L_* of L^-^ | 0.40/h * |  |
| *K_I_* | Isoleucine saturation constant of I^-^ | 0.048 μM * | 1 μM ^¶^ |
| *K_L_* | Leucine saturation constant of L^-^ | 0.038 μM * | 10 μM ^¶^ |
| *K_S_* | Glucose saturation constant of both strain | 1.0 μM * |  |
| *m_I_* | Maintenance rate of I^-^ | 0.038/h ^§^ |  |
| *m_L_* | Maintenance rate of L^-^ | 0.026/h ^§^ |  |
| *a_L_* | Number of L^-^ cells produced by leucine from a single I^-^ cell until its death | 0.70 cell/cell ^†^ |  |
| *k_L_* | Rate of isoleucine supply (I^-^ cell equivalent) by a single L^-^ cell | 0.0071/h ^‡^ | 0.4/h ^#^ |
| *c_I_* | Number of I^-^ cells produced by a unit of isoleucine | 0.98 cell/(fmol) * |  |
| *c_L_* | Number of L^-^ cells produced by a unit of leucine | 1.8 cell/(fmol) * |  |
| *c_S_* | Number of cells of both strain produced by a unit of glucose | 0.019 cell/(fmol) * |  |

Value_mono_ is the value obtained from the monoculture experiments. For the values of *K_I_*, *K_L_* and *k_L_*, we used different values from the Value_mono_ for the simulation (Value_co_) as the values for coculture. For the simulation shown in Figs. S1B and S1C, the values were used as one significant figure.

* From the growth curve and the saturation concentration of I^–^ or L^–^ cells when various concentrations of nutrient were supplied. Values of *μ_I_*, *μ_L_*, *K_I_*, *K_L_* and *K_S_* were obtained from the fitting of the growth rate in the growth phase to the modified Monod equation (equation 5 in (1)) using the values of the maintenance rates. Values of *c_I_*, *c_L_* and *c_S_* were obtained from the correlation between the concentration of the supplied nutrient and the saturation concentration of the cells.

§ Obtained from the fitting of the decrease in the cfu to the single exponential in Figs. S5B and S5C.

† Obtained from the ratio of the concentration of Leu to that of I^–^ cells at the final couple of data points in Fig. 1B.

‡ Obtained from the ratio of the initial slope (0~21 h) of the Ile concentration to the L^–^ cell concentration (averaged for 0~21 h) in Fig. 1E. This value can also explain the results shown in Fig. 1D. The number of I^–^ cells produced from the Ile supplement from a single new L^–^ cell until its death (*k_L_*/*m_L_*) was 0.27, which is roughly consistent with the ratio of the concentration of Ile to that of L^–^ cells at the final point in Figs. 1D and 1E.

# Obtained from the ratio of the initial slope (0~22 h) of the I^–^ concentration to the L^–^ concentration (averaged for 0~22 h) in Fig. 3D. *k_L_*/*m_L_* was 13, which is roughly consistent with the ratio of the concentration of I^–^ cells to that of L^–^ cells after initial I^–^ cell growth in coculture, as shown by the arrows in Fig. 2.

¶ Estimated from a comparison of the simulation results with the experimental results in Fig. 2 by varying each value at intervals of one order of magnitude.

1. Kovarova-Kovar K & Egli T (1998) Growth kinetics of suspended microbial cells: from single-substrate-controlled growth to mixed-substrate kinetics. *Microbiol Mol Biol Rev* 62(3):646-666.
